# Mid-term outcomes after single anastomosis sleeve ileal (SASI) bypass in treatment of morbid obesity

**DOI:** 10.1007/s00464-023-10112-y

**Published:** 2023-05-12

**Authors:** Ebrahim Aghajani, Carl Schou, Hjortur Gislason, Bent Johnny Nergaard

**Affiliations:** 1 Department of Surgery, Aleris Obesity Clinic, Aleris Hospital, Fredriks Stangs Gate 11-13, 0246 Oslo, Norway; 2 Metabolic and Bariatric Unit, GB Obesitas, Skeppsbron 11, 211 20 Malmo, Sweden

**Keywords:** Bariatric surgery, Single anastomosis sleeve ileal bypass, SASI, Obesity, Diabetes, Metabolic surgery

## Abstract

**Background:**

According to several short-term studies, single-anastomosis sleeve ileal (SASI) bypass offers similar weight loss to Roux-en-Y Gastric Bypass (RYGB) with fewer complication and better comorbidity reduction/resolution. Long-term data on this operation is lacking in the literature. The purpose of this study was to analyze the outcomes of SASI bypass up to 4 years.

**Methods:**

This study is a retrospective cohort analysis of 366 patients with morbid obesity who underwent primary SASI bypass from January 2018 to February 2022.

**Results:**

The mean age and preoperative body mass index (BMI) were 41 years (range 22–71 years) and 43.9 ± 6.5 kg/m^2^, respectively. Follow-up was available for 229 patients at 1-year (89%), 112 patients at 2-year (75%), 61 patients at 3-year (75%), and 35 patients at 4-year (71%). The intraoperative, short-term, and long-term complication rates were 0%, 2.5%, 4.6%, respectively. After 4 years, mean percentage excess weight loss (%EWL) was 93.3% and total weight loss (%TWL) was 41.2%. Remission of comorbidities was 93% for type 2 diabetes mellitus, 73% for hypertension, 83% for hyperlipidemia, 79% for sleep apnea, and 25% for gastroesophageal reflux disease (GERD). Biliary gastritis and ulcers are seldom. Eight patients developed de novo GERD symptoms requiring proton pump inhibitor treatment. None of the patients in our study had hypoalbuminemia or malabsorption that did not respond to increased protein intake and vitamin or mineral supplementation.

**Conclusion:**

SASI bypass appears to be safe, and one of the most effective bariatric procedures regarding weight loss and obesity related comorbidities. The double-outlet created in this procedure seemingly minimizes nutritional complications.

**Graphical abstract:**

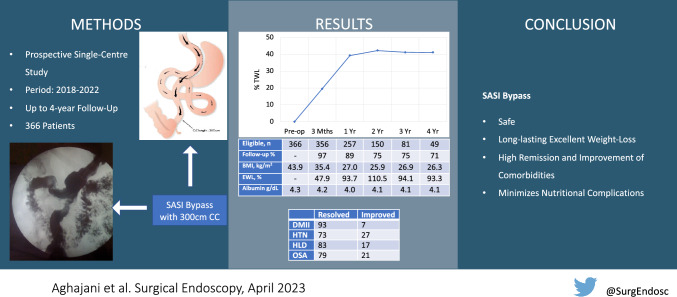

Obesity is an increasingly problematic global health issue, the World health organization, WHO, reports that the worldwide obesity has nearly tripled since 1975 [1]. Scandinavian countries are no exception to this challenge; today, about 70 per cent of Norway’s population is overweight or obese. Bariatric and metabolic surgery are the most efficient treatments for obesity and its related morbidities [2]. Currently, the Roux-en-Y Gastric Bypass (RYGB) and the One-Anastomosis Gastric Bypass (OAGB) are two of the three most common bariatric procedures [3]. Both procedures have several drawbacks, such as malabsorption and the resulting consequences that follow an exclusion of the duodenum.

Sleeve gastrectomy with transit bipartition (SG + TB) was initially described by Santoro in 2012 [4]. A modified SG + TB performing a loop rather than a Roux-en-Y bipartition reconstruction was coined “SASI bypass” by Mahdy and Mui [5, 6]. Comparatively to SG + TB, loop-anastomosis is preferable in being a more straightforward operation that minimizes the risk of internal hernias and is easily convertible into other bariatric procedures.

Recent data proposes that the SASI bypass is at least as efficient in treating weight loss and comorbidities as the RYGB, with the advantage of being less technically difficult and morbid [7–11]. In order to draw definite conclusions regarding the procedure, a more extensive series with a longer follow-up is necessary. Our study contains a large sample of patients undergoing SASI bypass with the longest follow-up to date.

## Methods

This study is a retrospective analysis of prospectively collected data on 366 patients with morbid obesity who underwent SASI bypass (primary operation) from January 2018 to February 2022 in our hospital.

A multidisciplinary approach from surgeons, nurses and dieticians followed the patient’s first educational seminar. During the seminar, patients were informed about the various surgical procedures. After this, the patients had an individual evaluation in the clinic. Each patient signed an informed consent for surgery and participation in this study, detailing the surgery, potential risks, and benefits. All patients who underwent SASI bypass were informed about the experimental nature of the procedure and the lack of available data.

Ethical approval for the study was obtained from the Regional Committee for Medical Research Ethics (66095).

Clinical data was prospectively collected and registered in our database (FileMaker 11) as part of our routine patient record system, approved by The Norwegian Data Protection. Patients with BMI over 40 or with BMI over 35 with comorbidities were offered SASI bypass with 300 cm common limb. All data regarding weight loss, metabolic status, postoperative changes, and complications were registered continuously. Follow-up is performed via visits at 3 and 12 months and then once annually.

The resolutions or improvements of type 2 diabetes (T2D), hypertension (HTN), hyperlipidemia (HLD), and obstructive sleep apnea (OSA) were defined according to the American Society for Metabolic and Bariatric Surgery guidelines [12]. Gastroesophageal reflux disease (GERD) was considered as typical reflux symptoms with a need for proton pump inhibitor (PPI) treatment at least 1 day a week. Furthermore, complete remission was considered as absence of symptoms and discontinuation of PPI.

Insulin resistance was assessed by the Homeostasis Model Assessment of Insulin Resistance (HOMA-IR) with the formula: fasting glucose (mmol/L) × fasting insulin (mIU/L)/22.5. Patients receiving insulin treatment were excluded from the analysis of insulin and HOMA-IR. The short-term (< 30 days) and long-term (> 30 days) complications were reviewed and graded on the Clavien-Dindo scale [13].

### Statistical analysis

All calculations were analyzed using IBM SPSS version 24 for Windows. Continuous variables were characterized using means and standard deviations. Categoric variables were characterized using frequencies and percentages. Continuous variables were compared using paired t test. For nutritional data, Fisher`s exact test was used. A p value of < 0.05 was considered statistically significant.

### Operative technique

Our technique is the same as reported by Mahdy [6], with some technical differences. Briefly, the laparoscopic sleeve gastrectomy (SG) is created by stapling alongside a 32 Fr. bougie on the lesser curvature. The staple line in all patients starts approximately 5 cm from the pylorus and ends at the angle of his and includes the complete fundus. The terminal ileum is identified and measured proximally up to around 300 cm. The loop was ascended antecolic and stapled linearly, isoperistaltic side-to-side to the anterior wall of the gastric antrum 2–5 cm away from the pylorus. Crucially, we attempt to create an anastomosis size of between 2.5 and 3 cm. The defect of the gastroileal anastomosis is closed with a continuous running suture. A methylene blue test is performed to assess the presence of any leaks. An anti-tension row is sewn between the afferent-loop and 5 cm of the sleeved staple line.

All subjects followed the same postoperative protocol: a liquid diet for two weeks, a soft diet for the following two weeks, the use of low-molecular-weight heparins for two weeks, and the use of pantoprazole for three months to prevent marginal ulcers. These daily oral supplements include: Multivitamin, 1000 μg of vitamin B12, 1000 mg calcium carbonate, 40 μg Vitamin D3, and 65 mg iron in fertile women. During follow-up, the dose of these supplements may need to be adjusted. Life-long vitamin supplementation is advised.

**Fig. 1:**
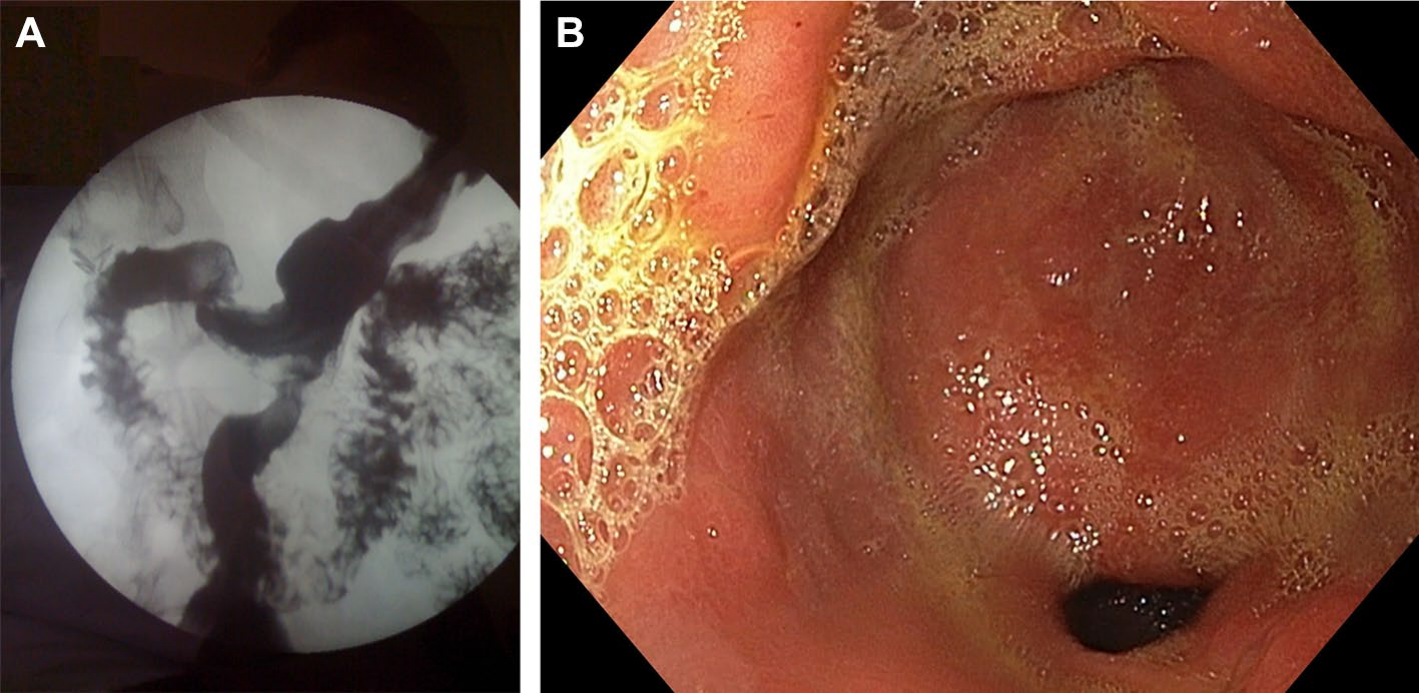
**a** Gastrografin (left) and **b** Upper endoscopy study (right) 12 months after SASI bypass

At the 1-year follow-up, gastrografin swallow test and upper endoscopy were routinely performed in the first 20 patients (Fig. 1a, b) to confirm a two-way passage and to exclude any biliary gastritis, ulcers, or stricture of anastomosis.

## Results

Three hundred sixty-six patients (285 females and 81 males) with a mean age of 41 years (range 22–71 years) who underwent SASI bypass as a primary procedure between January 2018 and February 2022 were analyzed. The demographic and clinical characteristics of the patients are provided in Table 1. Of 366 patients, 257, 150, 81, and 49 were beyond the 1, 2, 3, and 4-year postoperative mark, respectively. However, follow-up was only possible for 229 patients at 1-year (89%), 112 patients at 2-year (75%), 61 patients at 3-year (75%), and 35 patients at 4-year (71%). The 3-month follow-up was 97%. A laparoscopic approach was successfully completed in all patients without conversion. The mean operative time was 61 ± 14 min, with a mean length of stay at 1.3 ± 0.7. The intraoperative complication rate was 0%.

**Table 1:** Preoperative characteristics of patients

Variables	Value
Age at operation, years^a^	41 (18–71)
Sex	
Female	285 (78%)
Male	81 (22%)
BMI at operation (Kg/m^2^)	43.9 ± 6.5
Diabetic state	
None but with Insulin resistance (%)	287 (78.4%)
Type 2 diabetes mellitus (%)	36 (9.8%)
Hypertension (%)	80 (22%)
Hyperlipidemia (%)	260 (71%)
Obstructive sleep apnea (%)	36 (9.8%)
Gastroesophageal reflux disease (%)	65 (17.8%)

^a^Value expressed as mean and range

### Short-term and long-term complications

The short-term (< 30 days) complication rate was 2.5% (Table 2). Complications included one incident of pulmonary embolism, which responded to conservative treatment, and one trocar site hernia, which was managed laparoscopically. Further, four patients had intraabdominal bleeding, which required laparoscopic exploration with hematoma evacuation. No definite bleeding source was detected in these patients, and conservative measures, including blood transfusion, managed the condition. Intraluminal bleeding occurred in one case; the patient presented with hematemesis and rectal bleeding on the second postoperative day. The bleeding was managed endoscopically with an adrenaline injection. Lastly, an instant operation due to a leak at the gastroileal anastomosis site was necessary in one patient; this case was further complicated by sepsis, requiring ICU treatment for a prolonged period.

**Table 2:** 30-day results and early complications according to Clavin Dindo

Variables	Value (n = 366)	
Operative time, min	61 ± 14	
Length of hospital stay, day	1.3 ± 0.7	
Complications		Grade
Anastomotic leakage	1	IV
Intra-abdominal bleeding	4	III
Intraluminal bleeding	1	III
Trocar site hernia	1	III
Pulmonary embolism	1	II
Pneumonia	1	II
Total, n (%)	9 (2.5)	

The long-term (> 30 days) complication rate was 4.6% (Table 3). Stenosis at the gastroileal anastomoses had appeared in 4 patients, and a stomal ulcer had appeared in one patient (smoker); these were treated successfully with laparoscopic anastomosis revisions.

**Table 3:** Long term complications

Variable	Incidents	Grade of complication
Gallstones	9	Grade III
Stomal ulcer	1	Grade III
Anastomotic stricture	4	Grade III
Diarrhea	2	Grade III
Chronic constipation	1	Grade III
Total, n (%)	17 (4.6)	

Gallstones developed in 9 patients and were managed with laparoscopic cholecystectomy. Three patients had reversal operations to the SG, one at six months postoperatively because of constipation, and two patients due to excessive weight loss (EWL > 100%) and diarrhea in their second year after surgery.

### Weight loss outcome and nutrition status

**Fig. 2:**
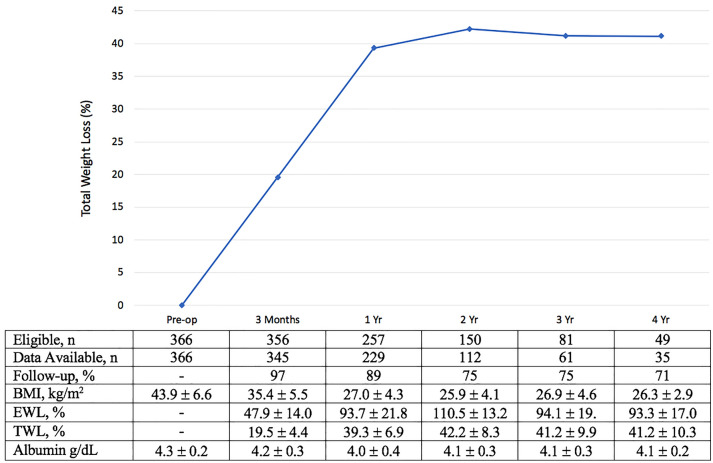
Weight loss outcomes and Albumin through 4 years after SASI bypass

Figure 2 depicts the percentage of total weight loss (%TWL) postoperatively.

**Fig. 3:**
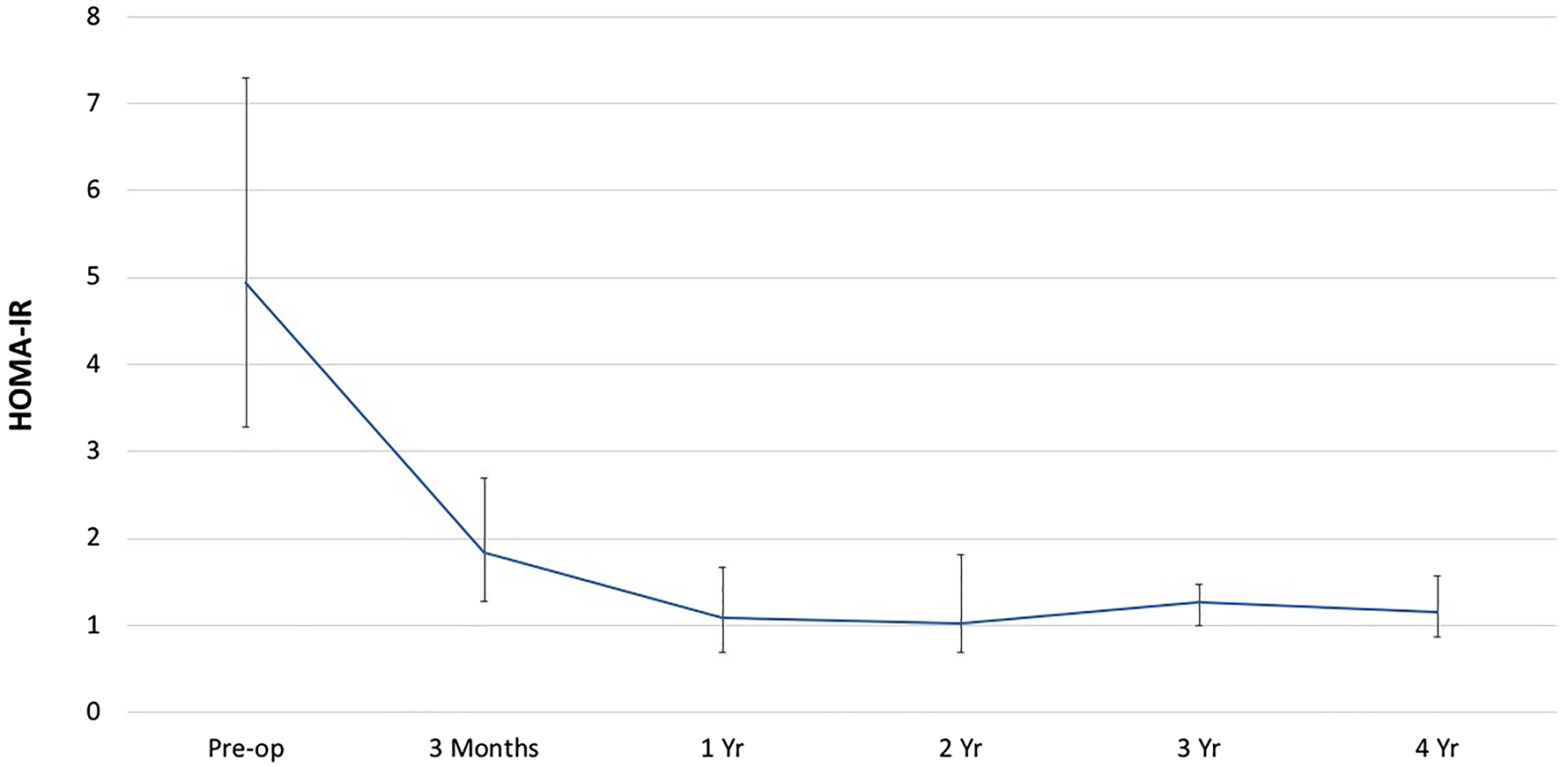
Median HOMA-IR through 4 years after SASI bypass with interquartile range

The mean BMI at 1, 2, and 4-year marks were 27.0 ± 4.3, 25.9 ± 4.1, and 26.3 ± 2.9 kg/m2, with a mean percentage of excess weight loss (%EWL) of 93.7%, 110.5%, and 93.3% respectively. The 1, 2, and 4-year %TWL was 39.3%, 42.2%, and 41.2%, respectively. Comparatively to the baseline, at the 3-year mark, insulin, HbA1c, and HOMA-IR levels improved significantly (Table 4 and Fig. 3). Lipid metabolism improved postoperatively.

**Table 4:** Nutritional outcomes with SASI bypass at 3 years

	Preoperative (n = 366)		Normal range	3 years (N = 61)		P value
	Mean ± SD	Abnormal (%)		Mean ± SD	Abnormal (%)	
Glucose	99.8 ± 21.9	34 (9.4)	65–100 mg/dL	84.8 ± 7.9	2 (4)	< 0.05
HbA1C	5.6 ± 0.8	24 (7.2)	4–6%	5.1 ± 0.4	1 (2)	< 0.05
Insulin	24.5 ± 14.7	92 (26)	2–25 mIU/L	7.9 ± 6.5	1 (2.4)	< 0.05
Hb^a^	14.1 ± 1.13	5 (1.3)	11.7–15.3/17 g/dL	13.2 ± 1.3	3 (6)	< 0.05
Ca	9.2 ± 0.36	15 (4)	8.6–10.2 mg/dL	9.1 ± 0.4	3 (6)	ns
PTH	78.4 ± 29.4	67 (19)	11–79 pg/mL	69.3 ± 30.1	9 (1.8)	ns
Albumin	4.3 ± 0.2	4 (1)	3.6–4.8 g/dL	4.1 ± 0.3	2 (4)	ns
TG	154.9 ± 79.6	104 (28.7)	< 150 mg/dL	79.7 ± 53.1	1 (2)	< 0.05
Cholesterol	193.3 ± 36	25 (6.2)	100–199 mg/dL	158 ± 36	4 (8)	< 0.05
Ferritin^b^	132.6 ± 114	33 (9)	12–150/300 ng/mL	87.8 ± 98.7	8 (16)	< 0.05
Vit B12	451.2 ± 195	12 (3.2)	230–880 pg/mL	853.1 ± 442.9	18 (37)	< 0.05
Vit D	23.2 ± 9.7	294 (82)	32–100 ng/mL	34.7 ± 12.1	5 (11)	< 0.05
Zink	92.1 ± 10.4	10 (3)	58–111 μg/dL	80.4 ± 13.7	2 (5.1)	ns

*HbA1C* hemoglobin A1C, *Ca* calcium, *PTH* parathyroid hormone, *TG* triglyceride

^a^Hb value for males 13.4–17.0 and females 11.7–15.3 g/dL

^b^Ferritin value for males 20–300, and females 15–200 ng/mL

The Albumin levels remained within normal range postoperatively (Fig. 2). Vitamin D deficiency was present preoperatively in many patients and, like vitamin B12, increased after the operation with supplementation. Despite a slight difference in serum Ca, Zink, and PTH at the 3-year mark compared to the preoperative value, they were still within the normal range. Three patients had mild anemia (Hb = 10.5–11.7 g/dL), and eight had low ferritin at the 3-year follow-up. Most of these patients had menorrhagia. Ferritin and Hb levels eventually normalized with extra iron supplementation and reduction of menorrhagia.

### Effects on obesity-related comorbidities

Over four years, complete remission of T2D, HTN, HLD, OSA, and GERD took place in 93%, 73%, 83%, 79% and 25% of patients, respectively (Table 5). Improvement of T2D, HTN, HLD, OSA, and GERD appeared in 7%, 27%, 17%, 21%, and 29%, respectively. Preoperative GERD remained stationary in 46% of patients. Eight patients developed de novo GERD symptoms. HOMA-IR index levels decreased significantly after the third month of surgery and reached a platform stage (Fig. 3).

**Table 5:** Remissions of comorbidities through 4 years after SASI bypass

Comorbidities	Preoperative, %	Postoperative, %			
		Resolved	Improved	Neutral	Worsened
Type 2 diabetes mellitus	9.8	93	7	0	0
Hypertension	21.9	73	27	0	0
Hyperlipidemia	71	83	17	0	0
Obstructive sleep apnea	9.8	79	21	0	0
GERD	17.8	25	29	46	0

## Discussion

The search for the best bariatric procedure continues. Presently, SG is the most commonly performed bariatric procedure globally [3]. However, this procedure has several drawbacks, such as insufficient weight loss, weight regain, and GERD [14, 15].

SG + TB and SASI bypass are based on modulating the neuroendocrine control of hunger and satiety, not malabsorption. The SG decreases ghrelin secretion by removing most of the gastric parietal mass. The rationale of gastroileal anastomosis is the rapid flow of most food into the distal intestine, causing a more effective hindgut stimulation, thereby, more effective secretion of GLP-1 and PYY. These hormones reduce the rate of gastric emptying (functional restriction) and increase insulin secretion. Both procedures maintain, at least in part, gastric digestion, pyloric function and all duodenal, jejunal and ileal physiology without any degree of clinically detectable malabsorption. Recent review articles [16, 17] strongly support the recommendation of SASI bypass more than other bariatric operations. However, most current evidence consists of small to medium-sized cohort studies with short follow-ups. Recently, Sewefy et al. published the long-term results (> 5 years) of a large cohort of single anastomosis sleeve jejunal (SASJ) bypass, which is a modification of SASI bypass with a shorter biliopancreatic limb length [18]. Our study reports a large bariatric sample of patients undergoing SASI bypass with the most extended follow-up to date.

The weight loss was excellent from the beginning postoperatively. The %EWL was 48% by the first three postoperative months, reaching a mean value of 97% at 12 months postoperatively. In addition to weight loss, HTN, HLD, OSA and GERD remissions and improvements were observed in most patients (Table 5). The metabolic benefits in patients with T2D were quite convincing, with marked improvements in fasting insulin, glucose, and HbA1c levels and a reduction in insulin resistance. The %TWL, and the rate of remission or improvement of the obesity-related comorbidities are in accordance with other SASI studies [7–10, 18–22], and higher than that of other bariatric procedures [14, 15, 23].

The remission or improvement of GERD may be related to the effect of gastroilostomy, which may reduce intra-gastric pressure and accelerate gastric emptying. All eight patients with de novo reflux have acceptable sporadic reflux with a PPI therapy of 40 mg daily. Routine upper endoscopy in the first 20 patients at 1-year follow-up and four patients with de novo GERD were completely normal (Fig. 1b). In terms of improvement of GERD, SASI bypass have superior results to SG [19]. In addition, in the case of uncontrolled GERD, despite PPI therapy, it can easily be converted to RYGB.

We report a relatively low incidence (2.2%) of de novo GERD in our study. This is compared to some sleeve studies with incidence of up to 25% [14, 24]. Our definition of reflux disease was based on patient history like typical reflux symptoms and daily intake of PPI. Some patients presented a history of severe reflux disease based on endoscopic findings with oesophagitis grade C and hiatal hernia. These patients were advised to a standard RYGB. No patients in our study had hiatal hernia. However, if patients were to present with intraoperative findings of hiatal hernia and symptomatic GERD, our strategy includes different anti-reflux operations such as simple posterior hiatoplasty with cardiopexy or GS with Toupet fundoplication. Our results regarding GERD and de novo reflux provide only a symptomatic picture of GERD, before and after SASI bypass, and is not sufficient to draw any firm conclusions.

The reported short- and long-term complication rates, 2.5% and 4.6%, respectively, were within established reports, and no mortality occurred.

Notably, some studies have indicated a reversal of SASI bypass to SG due to hypoalbuminemia or %EWL > 100% [6, 8, 9]. None of the patients in our study had hypoalbuminemia or malabsorption (Fig. 2) that did not respond to increased protein intake and vitamin or mineral supplementation. This is in accordance with other studies [6, 20, 21]. In the present study, three patients were revised to SG for different reasons (Table 3). These patients had been operated on at the beginning of this study; today, we believe that these revisions could have been avoided with conservative treatment and close dietetic follow-up from nutritionists. We chose the common limb length (300 cm from ileocecal) from our experience of different limb lengths in RYGB procedures [25, 26]. A rule of thumb for any malabsorptive procedure should be maintaining at least 300 cm of the small bowel in the food stream to prevent the development of malnutrition. Longer common channel length and increased focus on dietetic follow-up may explain this study's lower incidence of diarrhea and malabsorptive issues. In SASI bypass, the limb length can be adjusted to reduce nutritional problems or converted easily to other procedures if needed [27, 28]. Like all other metabolic surgeries, vitamin and mineral supplementation is strongly encouraged after SASI bypass [29].

The major advantage of a single-loop anastomosis of SASI bypass over a double anastomosis in SG + TB is a simpler procedure, shorter operative time and less risk of anastomosis complications and internal hernia. There is always a risk of Petersen's defect hernia in SASI bypass, like with other single anastomosis procedures, namely, OAGB, single anastomosis duodenal-ileal bypass with sleeve (SADI-S). However, the risk of strangulation is lower than possible complications with routine closure, such as mesenteric hematoma or incomplete closure [30]. We did not experience any internal herniation in this study, although we did not routinely close the Petersen space.

The limitations of the present study include the retrospective nature of the study and relatively healthy patients. Most of the patients in our study are young with few obesity-related comorbidities but with high insulin resistance (78.4% of non-diabetic). Weight loss-independent reduction in insulin resistance within three months postoperative shows the profound metabolic effect of SASI bypass. With the knowledge of the importance of insulin resistance for the development of metabolic syndrome, prevention of this risk factor has not only very high individual health benefits but also societal benefits for its potential reduction in future metabolic syndrome costs.

The other limitation is the sample size and that only a small percent of the total sample size was available for a follow-up at the 4-year mark. Longer-term outcomes in additional cohorts are needed to evaluate the durability of these results.

## Conclusions

SASI bypass appears to be safe, and one of the most effective bariatric procedures regarding weight loss and obesity related comorbidities. The double-outlet created in this procedure seemingly minimizes nutritional complications and reduces the risk of developing GERD.
